# How Do Viruses Interact with Stress-Associated RNA Granules?

**DOI:** 10.1371/journal.ppat.1002741

**Published:** 2012-06-28

**Authors:** Richard E. Lloyd

**Affiliations:** Department of Molecular Virology and Microbiology, Baylor College of Medicine, Houston, Texas, United States of America; Columbia University, United States of America

## The Stress of Virus Infections Activates Cellular Stress Responses

**Figure 1:**
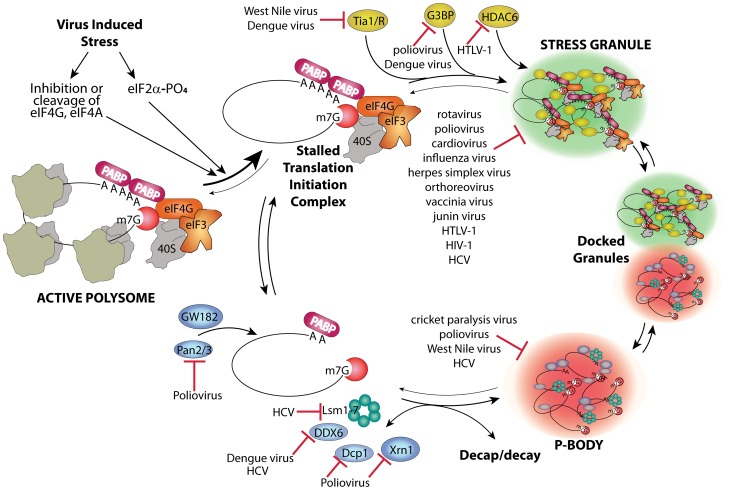
Stress granule and P-body assembly and interference by viruses. Virus infection causes stress at multiple levels that reduces host translation through activation of eIF2 kinases or other means and converts active polysome mRNPs into stalled translation initiation complex mRNPs. A complex series of events involving nucleation of several stress granule marker proteins such as G3BP, Tia-1/TIAR, and HDAC6 plus transport on microtubules (MT) leads to aggregates of translation initiation complex mRNPs in stress granules. Alternatively, mRNPs can be stripped of initiation factors and ribosome subunits, associate with GW182, undergo Pan2/3-mediated deadenylation, MT transport, and association of other RNA decay factors (e.g., Xrn1, Dcp1a, DDX6, GW182 and Lsm components of the exosome), and become concentrated in P-bodies. Decapping and decay occur outside P-bodies and also within them. Specific points/proteins where viruses interact with and inhibit RNA granule assembly pathways are shown.

Host mRNAs are always dynamically exchanged between translating and non-translating pools. Non-translating pools are organized into specialized RNA granules called stress granules (SGs) and processing bodies (P-bodies, PBs), which have fundamental roles in inhibition and degradation of host mRNAs (Figure 1) [1]. Virus infection usually results in interference in many cell processes in ways that directly induce stress responses. Cells respond to many types of stress by transient global inhibition of protein synthesis in order to promote cell survival through restricted consumption of nutrients and energy. This can also redirect gene expression and resources to damage repair pathways. The most common form of global translation arrest comes by restricting production of ternary complex consisting of eukaryotic initiation factor 2(eIF2)•GTP•met-tRNA_i_^met^, which must bind 40 S ribosome subunits to facilitate mRNA scanning and start codon selection at the initiation step. Restriction of ternary complex formation is accomplished by phosphorylation of the alpha subunit of eIF2 by one of four conserved eIF2α kinases that sense various types of cell stress [2]. Restriction of translation from activation of eIF2 kinases results in accumulation of stalled preinitiation complexes containing 40 S ribosomal subunits. After translation repression by this means, or alternate mechanisms such as cleavage of eIF4G scaffold protein or inhibition of eIF4E helicase, cells respond by organizing mRNPs with stalled translation initiation complexes into SG foci. The mechanism of SG formation is poorly understood but involves mRNP remodeling that incorporates new proteins that may nucleate SGs and involves mRNP transport on microtubules (Figure 1). SGs may facilitate rapid reactivation of translation upon stress recovery since ribosome preinitiation complexes are retained in an assembled state. SGs may also promote cell survival during stress since they sequester components of apoptotic signal transduction pathways such as RACK1 [3].

SGs and PBs have fundamental roles in inhibition and degradation of host mRNAs, and thus will affect the metabolic fate of viral mRNAs. Viruses interfere with the cellular gene expression machinery, thus it is no surprise that many viruses interact in different ways with both SG and PB responses and components to control virus replication and antiviral responses. Although SG formation is frequently induced by virus infection, many differences exist in the dynamics and outcome of the stress responses induced by various viruses.

## Viruses Control SG Formation

No virus infection can succeed if viral mRNA is sequestered into translationally silenced mRNPs that aggregate in SG structures. Thus, viruses have evolved counter measures to prevent this fate. Species from many virus families can be organized into groups based on how they repress SGs. For instance, one group of viruses transiently triggers SG formation early in replicative cycles but restricts SGs later. Many of these viruses, such as poliovirus, alphavirus, and orthoreovirus, have replication cycles that activate eIF2α kinases [4]; however, mammalian orthoreoviruses can induce SG by viral entry alone [5]. This basic host–virus relationship is conserved in nature since insect dicistrovirus also antagonizes SG formation [6]. Another group of viruses effectively represses SG formation throughout infection, which is only revealed when their defective mutant viruses induce SGs or SG-like structures. These include herpes simplex virus mutant lacking the vhs host shutoff gene [7] and influenza virus with NS1 mutants [8].

Many viruses within the above groups overtly block the host cell's ability to form SGs at some point during infection. This is often measured by loss of cellular SG formation in response to oxidative stress from arsenite treatment, which is the most accepted standard for canonical SG formation. Viruses that block this response include poliovirus, orthoreovirus, human immunodeficiency virus 1 (HIV-1), cardiovirus, junin virus, and rotavirus (reviewed in [4]). Viral gene expression leads to suppression of the cellular SG response through an expanding variety of mechanisms. Only a few have been elucidated in any detail, but one common pattern emerging is destruction or sequestration of key host factors required for SG formation. For instance, poliovirus 3C protease cleaves the key SG-nucleating protein G3BP [9], which prevents colocalization of initiation factors, ribosome subunits, and mRNA in silenced SG foci. Another SG-nucleating protein, TIA1, continues to aggregate after G3BP cleavage [10], but only in smaller vestigial foci that are stripped of initiation factors and most mRNA [11]. The flaviviruses West Nile virus and dengue virus sequester other SG-nucleating proteins, TIA1 and TIAR, in replication complexes [12], and dengue virus may also sequester SG proteins G3BP1, caprin1, and USP10 as well as PB marker protein DDX6 (also known as RCK/p54) [13]. HTLV-1 Tax protein interacts with HDAC-6, which is crucial for SG formation and maintenance [14]. Dicistrovirus also blocks formation of complete SGs by preventing inclusion of paralogs of TIA1 and G3BP [6]. Viruses that sequester key SG-nucleating components in novel aggregates may also be diverting SG assembly pathways to aid replication as discussed below.

## Viruses Can Disrupt PBs and RNA Decay Machinery

SGs are dynamically linked to PBs, which are another type of RNA granule packed with translationally silenced mRNPs and many enzymes of the RNA decay machinery [15]. SGs and PBs are thought to transiently bind and exchange mRNP cargo in association with remodeling of the mRNP protein constituents, and PBs may promote SG assembly [16], [17]. Since PBs are proposed to be sites where RNA decay of translationally repressed mRNA occurs, it is likely that viruses will antagonize PB functions that could otherwise lead to decay of viral mRNA. Indeed, West Nile virus and hepatitis C virus (HCV) infection leads to a progressive decline in PB foci after 24–36 h infection [12], [18]. HCV replication is stimulated by PB RNA helicase DDX3, which binds HCV core protein [19], and PB components Rck/p54, Lsm1, and PatL1 are required for HCV replication [20], [21]. Knockdown of certain PB components (Lsm1, DDX6) also reduced HCV replication, suggesting that PB components (as well as SG components) are required for HCV replication or assembly [18]. Some of these basic relationships are conserved in insect viruses since cricket paralysis virus partly disperses PB foci containing overexpressed marker proteins [6]. Poliovirus and coxsackievirus B3 also disrupt PBs, but much more aggressively, leading to total loss of PBs by 3–4 h after infection. In this case, virus infection results in cleavage or degradation of key components of the RNA decay pathway, Xrn1, Dcp1a, and Pan3, involved in both 5′ and 3′ mediated RNA decay [22]. Sindbis virus also antagonizes viral RNA decay by selective movement of HuR protein out of the nucleus where it binds and stabilizes viral transcripts [23]. HuR is known to antagonize inclusion of mRNA in PBs [24].

## Viruses Can Co-Opt Components of SGs and PBs for New Functions in Replication or Assembly

As canonical SGs do not commonly co-exist with active virus replication, it seems the overall effect of SGs on virus replication usually appears to be negative and is selected against. However, some viruses may co-opt and misdirect the SG response of cells to facilitate steps in virus replication. In this way the initial host response to stress could have a positive impact on certain virus infections, though significant aggregation of stalled translation initiation complexes is disallowed. For example, vaccinia virus (VV) may subvert SG-nucleating proteins and other constituents into novel aggregates that share some SG properties such as colocalized G3BP and initiation factors eIF4G and eIF4E but differ in that they contain no silenced mRNAs; rather, they contain VV mRNA instead. These structures form within and adjacent to viral replication factories and may help vaccinia segregate replication and packaging activities away from translation [25]. In an analogous way, HCV recruits several components of SGs to viral replication factories where they colocalize with HCV core protein. Recruited SG proteins include G3BP1, ataxin2, and PABP, which form alternative ring-like structures surrounding lipid droplets [18]. Finally, both HCV and dengue virus RNA are reported to bind G3BP [13], [26], and West Nile virus promotes plus strand RNA replication by using TIAR to bind a stem-loop structure on minus strand RNA templates [12], [27]. The interactions of SG-nucleating proteins with viral RNA and formation of novel viral foci containing SG proteins indicates viral foci may share common assembly mechanisms with SGs or PBs. The specific functions of redirected host proteins in virus replication or assembly have not yet been characterized.

## SGs and PBs May Function as Antiviral Components of Innate Immune Responses

Since many virus families repress SGs or PBs, these RNA granules may represent components of an integrated cellular stress response that has distinct antiviral properties. The formation of SGs is potentially antiviral on several functional levels. First, they sequester host translation initiation factors that may be limiting (e.g., eIF4E, eIF4G, eIF4A, eIF4B, and eIF3) and 40 S ribosome subunits, which are critical for viruses to translate their transcripts efficiently. Second, SGs sequester IRES transactivating factors (PTB, PCBP2, and UNR) required by classes of viruses (e.g., picornaviruses) for efficient IRES-mediated translation [11], [28]. Third, any other mRNA binding proteins that function in aspects of virus replication are likely to be concentrated in SGs as passengers on silenced mRNPs. As mentioned above, both HCV and West Nile virus apparently co-opt SG-nucleating proteins G3BP and TIA1/TIAR in or near replication complexes; thus, stable formation of functional SGs containing these factors may antagonize replication [12], [18]. Thus, the overall act of SG-mediated sequestration of needed factors away from general cytoplasmic pools can be viewed as generally antiviral.

In agreement with this, some experiments suggest that SG or at least SG components may function to repress productive virus infections. Expression of a cleavage-resistant mutant of G3BP that stabilized SGs against virus attack reduced replicative output of poliovirus [9]. Mouse embryo fibroblasts with TIA1 knocked out displayed increased virus production from several families of viruses, including vesicular stomatitis virus, Sindbis virus, and herpes simplex virus [27].

Formation of SG during infection could also sequester viral mRNA transcripts directly into these silenced non-translating pools, but interestingly, where this has been examined, there is no significant incorporation of viral mRNAs into SGs [10]. Possible exceptions are sequestration of HIV-1 transcripts via Nef mRNA interaction with SAM68 causing SG inclusion [29] and APOBEC3G protein binding to HIV RNA that may shunt RNA into SGs and PBs [30].

Apoptosis is another cell stress response that is largely antiviral in consequence; however, SG formation may signal survival and antagonize cell death. SG can block apoptosis by inhibiting the JNK/SAPK pathway via sequestration of RACK1 and other apoptosis-promoting factors into SG [3]. Thus, virus modulation of SG formation may require fine tuning to sequester sufficient pro-apoptotic factors while liberating sufficient pro-viral factors and translation apparatus to support efficient replication.

## Concluding Remarks

The complexity of virus–host relationships reveals common pathways and mechanisms, but also many exceptions that are virus-specific. This is evident in viral responses to SGs and PBs as viruses adapted their variable replication strategies to this common host impediment to replication.

It is also important to recognize that not all SGs are equivalent, as those induced by different stressors (e.g., heat shock) can contain some unique components that may influence their assembly or function [10]. Future work with viruses will reveal more mechanisms that can block SG formation, thus revealing novel insights into SG assembly. It will be interesting to learn how SG functions in integrated stress responses and if SG assembly itself further signals stress responses in forward feedback loops or antagonizes them in negative feedback loops, and to what extent crosstalk with Toll-like receptor or interferon signaling pathways occurs. Hopefully the lessons learned will translate into novel therapies to control viral gene expression and aid infection control and provide new insights into cancer and stress-associated degenerative diseases.
